# Cinacalcet-mediated activation of the CaMKKβ-LKB1-AMPK pathway attenuates diabetic nephropathy in *db/db* mice by modulation of apoptosis and autophagy

**DOI:** 10.1038/s41419-018-0324-4

**Published:** 2018-02-15

**Authors:** Ji Hee Lim, Hyung Wook Kim, Min Young Kim, Tae Woo Kim, Eun Nim Kim, Yaeni Kim, Sungjin Chung, Young Soo Kim, Bum Soon Choi, Yong-Soo Kim, Yoon Sik Chang, Hye Won Kim, Cheol Whee Park

**Affiliations:** 1 Division of Nephrology, Department of Internal Medicine, The Catholic University of Korea, Seoul, Korea; 2 Institute for Aging and Metabolic Diseases, College of Medicine, The Catholic University of Korea, Seoul, Korea; 3 Division of Nephrology, Department of Internal Medicine, St. Vincent’s Hospital, College of Medicine, The Catholic University of Korea, Suwon, Korea; 4 Department of Hematology, Seoul St. Mary’s Hospital, College of Medicine, The Catholic University of Korea, Seoul, Korea; 5 Department of Rehabilitation Medicine, College of Medicine, The Catholic University of Korea, Bucheon, Korea

## Abstract

Apoptosis and autophagy are harmoniously regulated biological processes for maintaining tissue homeostasis. AMP-activated protein kinase (AMPK) functions as a metabolic sensor to coordinate cellular survival and function in various organs, including the kidney. We investigated the renoprotective effects of cinacalcet in high-glucose treated human glomerular endothelial cells (HGECs), murine podocytes and C57BLKS/J-*db/db* mice. In cultured HGECs and podocytes, cinacalcet decreased oxidative stress and apoptosis and increased autophagy that were attributed to the increment of intracellular Ca^2+^ concentration and the phosphorylation of Ca^2+^/calmodulin-dependent protein kinase kinaseβ (CaMKKβ)-Liver kinase B1 (LKB1)-AMPK and their downstream signals including the phosphorylation of endothelial nitric oxide synthase (eNOS) and increases in superoxide dismutases and B cell leukemia/lymphoma 2/BCL-2-associated X protein expression. Interestingly, intracellular chelator BAPTA-AM reversed cinacalcet-induced CaMKKβ elevation and LKB1 phosphorylation. Cinacalcet reduced albuminuria without influencing either blood glucose or Ca^2+^ concentration and ameliorated diabetes-induced renal damage, which were related to the increased expression of calcium-sensing receptor and the phosphorylation of CaMKKβ-LKB1. Subsequent activation of AMPK was followed by the activation of peroxisome proliferator-activated receptor γ coactivator-1α and phospho-Ser^1177^eNOS-nitric oxide, resulting in a decrease in apoptosis and oxidative stress as well as an increase in autophagy.

Our results suggest that cinacalcet increases intracellular Ca^2+^ followed by an activation of CaMKKβ-LKB1-AMPK signaling in GECs and podocytes in the kidney, which provides a novel therapeutic means for type 2 diabetic nephropathy by modulation of apoptosis and autophagy.

## Introduction

Diabetic nephropathy (DN) is one of the severe forms of microvascular complication in type 2 diabetes that poses a major public-health burden worldwide^1^. Despite intense efforts in search for pharmacological therapies to deter the disease progression, the number of diabetic end stage renal disease patients continues to rise. Therefore, the development of a novel therapeutic agent is of paramount importance.

Calcium-sensing receptor (CaSR) belongs to the superfamily C of G protein-coupled receptors with seven transmembrane receptors. Cinacalcet is type II agonist that binds to the transmembrane domain and positively modulate CaSR. CaSRs are expressed in various kinds of cells including parathyroid hormone (PTH)-producing cells, vascular endothelial cells, smooth muscle cells, and renal tubular cells^2^. In the kidney, CaSR is expressed in many segments of the nephron^3^, including the proximal tubule^4^, cortical thick ascending limb of Henle^5^, inner medullar collecting duct^6^, and juxtaglomerular cells^7^. However, significant expression of CaSR in other parts of the kidney, either as RNA transcript or as protein, remained a matter of debate; While Riccardi et al^8^. reported *Casr* mRNA in glomeruli and in almost all other tubular segments, Yang et al^9^. demonstrated its expression only in distal tubular and cortical collecting ductal cells in glomeruli. The observed discrepancy on the expression of CaSR in the kidney may be attributable to the use of different CaSR probes with varying sensitivity and specificity^10^.

A decrease in the production or in the activity of nitric oxide (NO) results in the development of endothelial^11^ and podocyte dysfuction^12^ and has been proposed as one of the early signs of diabetic microangiopathy that contributes to the progression of DN^11^. Thus, pharmacological activation of the endothelial nitric oxide synthase (eNOS) pathway by way of ameliorating endothelial cell dysfunction has emerged as an attractive approach to prevent the disease progression^11,12^. Recently, CaSR was found to be expressed in immortalized endothelial cells of human aorta, and stimulation of the receptor by cinacalcet induced production of NO resulting in vasorelaxation^13^. Another study showed that calcimimetics significantly increased intracellular Ca^2+^ ([Ca^2+^]i) levels by mobilizing intracellular stores in human umbilical endothelial cell, which in turn augmented NO release by a time and Ca^2+^-dependent increase in eNOS-Ser^1177^ phosphorylation levels^14^. Taken together, these findings suggest that cinacalcet may play a vital role in the prevention of diabetic endothelial dysfunction via the activation of eNOS-NO pathway.

5′-AMP-activated protein kinase (AMPK) is a metabolic master-switch that regulates and maintains cellular energy homeostasis^15–17^. Loss of sensitivity of AMPK activation to cellular stress impairs metabolic regulation, increases oxidative stress and apoptosis, and reduces autophagic clearance^18^. AMPK is activated by 5′-AMP in three distinct ways, all of which are antagonized by high concentrations of ATP^19^. AMPK is activated by metabolic stresses that increases cellular ADP:ATP and/or AMP:ATP ratios by a mechanism requiring tumor suppressor liver kinase B1 (LKB1)^19^, Ca^2+^/calmodulin-dependent protein kinase kinaseβ (CaMKKβ) or transforming growth factor-β-activated kinase 1^20,21^. AMPK activation turns off mammalian target of rapamycin complex (mTORC)1 signaling which in turn diminishes mTORC1–dependent inhibitory phosphorylation on UNC51-like kinase (ULK)1 to allow ULK1-AMPK interaction for a net increase in AMPK activation, resulting in autophagy induction^22,23^. Our previous studies showed that AMPK activation also prevents renal lipotoxicity and inhibits renal cell glucotoxicity in a manner dependent on the activation of AMPK-silent information regulator T1 (SIRT1)-peroxisome proliferator-activated receptor γ coactivator-1α (PGC-1α) in type 2 diabetes^24,25^. A recent study also indicated that metformin, an AMPK activator, increases endothelial guanosine triphosphate cyclohydrolase, the rate-limiting enzyme in tetrahydrobiopterin (an essential cofactor for eNOS), resulting in improved endothelial dysfunction in DN^26^. Despite emerging significance on the role of AMPK activation in the diabetic kidney by altering paracrine communication between endothelial cells and podocytes, little is known about the roles of its upstream kinases such as CaMKKα/β and LKB1 in the regulation of AMPK.

Herein, we hypothesized that cinacalcet HCl directly modulates the upstream AMPK kinases such as [Ca^2+^]i, CaMKKα/β and/or LKB1, and the downstream signaling PGC-1α-eNOS-NO system to prevent and ameliorate DN in type 2 diabetes.

## Materials and methods

### Experimental mouse model

Eight-week-old male C57BLKS/J mice were purchased from Jackson Laboratories (Bar Harbor, ME). Male C57BLKS/J *db/m* and *db/db* mice were divided into four groups and received either a regular diet of chow or a diet containing cinacalcet (10 mg/kg, *n* = 8, respectively). Cinacalcet mixed with standard chow diet was fed for 12 weeks starting at 8 weeks of age. At week 20, all animals were anesthetized by intraperitoneal injection of 30 mg/kg tiletamine plus zolazepam (Zoletil; Virbac, Carros, France) and 10 mg/kg xylazine hydrochloride (Rompun; Bayer, Leuverkusen, Germany). Blood was collected from the left ventricle and the plasma was stored at −70 ℃ for subsequent analyses. All research procedures involving animals were performed in accordance with the Laboratory Animals Welfare Act, the Guide for the Care and Use of Laboratory Animals, and approved by the Institutional Animal Care and Use Committee at College of Medicine, the Catholic University of Korea.

### Assessment of albuminuria, urinary calcium, renal function, and oxidative stress

At week 20, the animals were housed in metabolic cages (Nalgene, Rochester, NY) for 24-h to collect urine for subsequent measurements of the albumin concentrations by an immunoassay (Bayer, Elkhart, IN). Plasma and urinary creatinine concentrations were measured using a HPLC (Beckman Instruments, Fullerton, CA). Plasma ionized calcium (iCa^2+^) PO_4_^−^ and urinary calcium concentrations were all measured by colorimetric assay (Samkwang Medical Laboratory, Seoul, Korea). To evaluate oxidative DNA damage and lipid peroxidation, we measured 24-h urinary 8-hydroxy-deoxyguanosine (8-OH-dG) and 8-epi-prostaglandin F_2α_ (isoprostane; OXIS Health Products, Portland, OR) concentrations, respectively.

### Histology

Kidneys were immersed in 10% formalin and embedded in paraffin. Histology was assessed by periodic-Schiff (PAS) staining. Mesangial matrix area and glomerular tuft area were quantified for each glomerular cross-section using PAS-staining sections. More than 30 glomeruli that were cut through the vascular pole were counted per kidney and the average was used for determination of the fractional mesangial area. We also performed immunohistochemistry for TGF-β1 (R&D Systems, Minneapolis, MN), type IV collagen (Biodesign International, Saco, ME) and cell surface glycoprotein F4/80 (Serotek, Oxford, UK) expression. All of these sections were examined in a blinded manner using light microscopy (Olympus BX-50, Olympus Optical, Tokyo, Japan). For the quantification of the proportional areas of staining, approximately 20 views (magnification) were used. These areas were randomly located in the renal cortex and the corticomedullary junction of each slide (Scion Image Beta 4.0.2, Frederik, MD). Apoptotic nuclei in tissue sections were detected using terminal deoxynucleotidyl transferase-mediated dUTP nick-end labeling (TUNEL) assay (Merck Millipore, Billerica, MA) and WT1 (Santa Cruz Biotechnology, Santa Cruz, CA). The TUNEL reaction was assessed in the whole glomeruli biopsy under magnification.We also performed immunofluorescence double staining for PECAM-1(Abcam, Cambridge, UK) and LC3B (Sigma-Aldrich, St. Louis, MO) and moreover nephrin (MyBioSource, San Diego, CA) and LC3B (Sigma-Aldrich). The fluorescent images were examined under a laser scanning confocal microscope system (Carl Zeiss LSM 700, Oberkochen, Germany).

### Western blot analysis

The total proteins of the renal cortical tissues were extracted with a Pro-Prep Protein Extraction Solution (Intron Biotechnology, Gyeonggi-Do, Korea), following the manufacturer instructions. Western blot was performed with specific antibodies for CaSR (Thermo Fisher Scientific Inc, Waltham, MA), CaMKKα/β (Santa Cruz Biotechnology), PGC-1α (Novus Biologicals, Littleton, CO), total LKB1(Cell Signaling Technology, Danvers, MA), phospho-Ser^428^ LKB1(Cell Signaling Technology), total AMPK (Cell Signaling Technology), phospho-Thr^172^ AMPK (Cell Signaling Technology), total eNOS (Cell Signaling Technology), phospho-Ser^1177^ eNOS (Cell Signaling Technology), B cell leukemia/lymphoma 2 (BCL-2), BCL-2-associated X protein (BAX) (Santa Cruz Biotechnology), Cu/Zn superoxide dismutase (SOD1) (Assay Designs, Ann Arbor, MI), Mn superoxide dismutase (SOD2) (Abcam), beclin-1 (Novus Biolobicals), LC3B (Sigma-Aldrich), tumor necrosis factor-α (Abcam), interleukin-1β (IL-1β) (Abcam), and β-actin (Sigma-Aldrich). After incubation with horseradish peroxidase-conjugated anti-mouse or anti-rabbit IgG (secondary antibody) (Cell Signaling Technology), target proteins were visualized by an enhanced chemiluminescence substrate (ECL Plus; GE. HealthcareBio-Science, Piscataway, NJ).

### Cell culture and small interfering RNA (siRNA) transfection

Human glomerular endothelial cells (HGECs) were purchased from Anigio-Proteomie (Boston, MA) and subcultured in endo-growth media (Angio-Proteomie, Boston, MA). We also cultured conditionally immortalized mouse podocytes were kindly provided by Dr. Peter Mundel (Albert Einstein College of Medicine, Bronx, NY) and were cultured as previously described^27^. The HGECs and podocytes were then exposed to low glucose or high glucose, with or without the additional 24-h administration of cinacalcet (1, 5, 15 nM). Western blotting was performed with specific antibodies for CaSR, CaMKKα/β, total LKB1, phospho-Ser^428^ LKB1, total AMPK, phospho-Thr^172^ AMPK, total eNOS, phospho-Ser^1177^ eNOS, BCL-2, BAX, SOD1, SOD2, and β-actin. siRNA, targeted to *CaMKKβ*, *LKB1*, *AMPKα1*, *AMPKα2*, and *SIRT1*, and scrambled siRNA (siRNAcont) were complexed with transfection reagent (Lipofectamin 2000; Invitrogen, Carlsbad, CA), according to the manufacturer’s instructions. The sequences of the siRNAs were as follows: CaMKKβ, 5′-GGAUCUGAUCAAAGGCAUCTT-3′; LKB1, 5′-GGACUGACGUGUAGAACAATT-3′; α1-AMPK, 5′-GCAUAUGCUGCAGGUAGAU-3′, α2-AMPK, 5′-CGUCAUUGAUGAUGAGGCU-3′ SIRT1, 5′-AAGACGGATTGCCCTCATTTG-3′ and nonspecific scrambled siRNA, 5′-CCUACGCCACCAAUUUCGU-3′ (Bioneer, Daejeon, Korea). HGECs in six-well plates were transfected with a final concentration of 50nM CaMKKβ, LKB1, α1 and α2-AMPK, and SIRT1 siRNAs for 24-h by transfection reagent (Lipofectamin 2000) in Opti-MEM medium (Gibco BRL, Grand Island, NY), according to the manufacturer instructions. Twenty-four hours after transfection, cells were treated with cinacalcet (5 nM) in high-glucose media to evaluate the effects of siRNA on HGECs.

### Intracellular Ca^2+^ ([Ca^2+^]i) measurement

Calcium concentrations were determined from the ratio of fura-2 fluorescence intensity at 340-nm excitation and 380-nm excitation. The 340-nm fluorescence of fura-2 increases and the 380-nm fluorescence decreases with increasing [Ca^2+^]i. For [Ca^2+^]i measurements HGECs and podocytes (20,000 cells/well) were plated on black 96-well plates with a clear bottom in complete medium. After 1 day the cells were serum-starved for 2-h. In the last 45 min of calcium-free serum-starvation, FURA-2AM (5 μM; Invitrogen) was added to the cells, then rinsed with Hanks Balanced Salt Solution (HBSS, Gibco BRL). FURA-2AM-loaded cells were sequentially excited at 340 and 380 nm by spectrophotometer microplate reader (Synergy MX; BioTek, Winooski, VT). Cinacalcet-induced [Ca^2+^]i were quantified by measurement of area under curve (AUC) and peak amplitude for the rise in relative [Ca^2+^]i. To evaluate cinacalcet-induced [Ca^2+^]i effects on CaMKKβ and LKB1, we used [Ca^2+^]i chelator BAPTA-AM (25 μM; Abcam).

### Immunofluorescence analysis

We performed immunofluorescence analysis for CasR, CaMKKα, CaMKKβ, pLKB1, pAMPK, and LC3 by using tyramide signal amplification fluorescence system (Perkin Elmer, Waltham, MA). Intracellular ROS levels were measured using the dihydroethidine (DHE, 2 μM, Invitrogen) assays. The proportion of apoptotic cells was determined using ApopTaq In Situ Apoptosis Detection kits (Merck Millipore, Billerica, MA), based on the TUNEL assay. Nuclei were stained by incubation with 4,6-diamidino2-phenylindole (DAPI). After mounting, fluorescence images were acquired using a confocal laser-scanning microscope (Carl Zeiss LSM 700, Oberkochen, Germany).

### Statistical analysis

The data were expressed as the mean ± standard deviation. Differences between the groups were examined for statistical significance using ANOVA with Bonferroni correction using SPSS version 19.0 (SPSS, Chicago, IL). A *P* value < 0.05 was considered statistically significant.

## Results

### **Increased [Ca**^**2+**^**]i by cinacalcet activates CaMKKβ-LKB1-AMPK-eNOS pathway in HGECs**

**Fig. 1:**
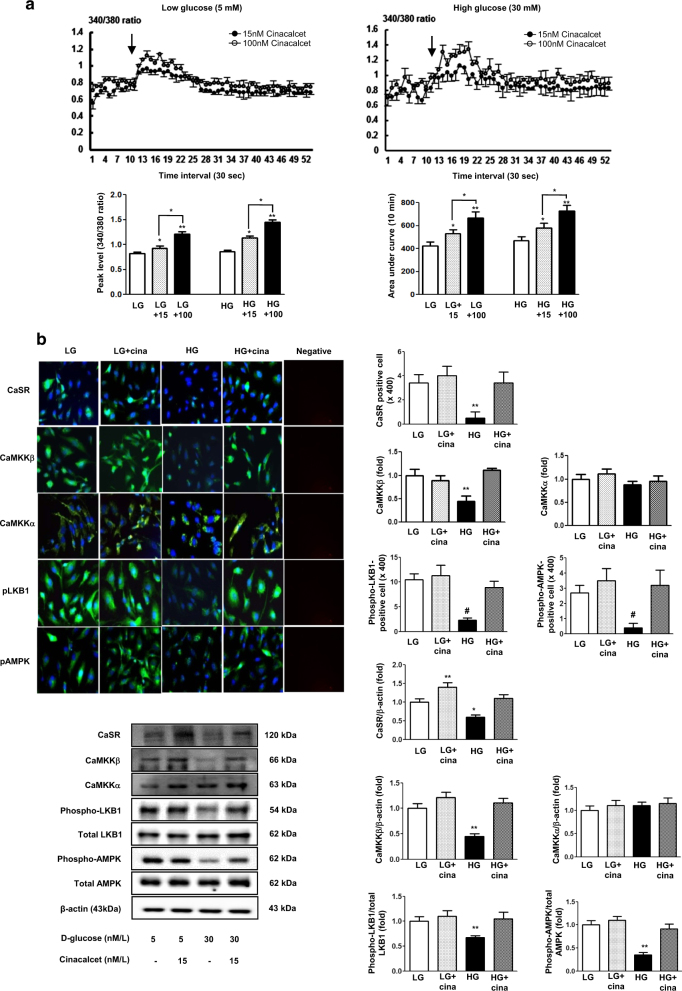
The changes of [Ca^2+^]i and intracellular signaling in HGECs exposed to cinacalcet and high-glucose media. **a** The changes of [Ca^2+^]i in HGECs exposed to cinacalcet and high-glucose media. To determine whether the addition of cinacalcet might modulate [Ca^2+^]i in HGECs, FURA-2AM-loaded HGECs were stimulated using different concentrations (15, 100 nM) of cinacalcet in low-glucose (LG; 5 mmol/l D-glucose) or high-glucose (HG; 30 mmol/l D-glucose) media. The area under curve (AUC) was estimated from the baseline of normalized data (at the point of injection) to a fluorescence level and between time points of injection (0 min) and 10 min. The peak of the curve was measured as highest value of the curve. The peak amplitude and AUC of [Ca^2+^]i were significantly increased by cinacalcet in dose-dependent manners in both LG and HG media. In Fig. 1a, the arrow denotes the administration of cinacalcet (15 and 100 nM, respectively) (*n* = 6 independent experiments in each experiments). *p < 0.05; **p < 0.01 compared with LG and HG. **b** The changes of intracellular signaling in HGECs exposed to cinacalcet and high-glucose media. Representative immunofluorescent (*n* = 6 independent experiments in each experiments) and western blot analyses (*n* = 4 independent experiments in each experiments) of CaSR, CaMKKα/β, phospho-Ser^428^ LKB1, and phospho-Thr^172^ AMPK in the cultured HGECs in low-glucose (LG; 5 mmol/l D-glucose) or high-glucose (HG; 30 mmol/l D-glucose) conditions with or without cinacalcet treatment (15 nM) and the quantitative analyses of the results are shown. *P < 0.05; **P < 0.01 and #*P* < 0.001 compared with other groups

To determine whether the addition of cinacalcet might modulate [Ca^2+^]i in our cellular model, FURA-2AM-loaded HGECs were stimulated using different concentrations (15, 100 nM) of cinacalcet in low and high-glucose media. As shown in Fig. 1a, cinacalcet markedly enhanced [Ca^2+^]i in low and high-glucose media in the absence of extracellular calcium. Interestingly, cinacalcet significantly increased the area under curve (AUC) and peak amplitude of [Ca^2+^]i in a dose-dependent manner in both low and high-glucose media. Thus the cinacalcet effect is independent of the glucose level.

**Fig. 2:**
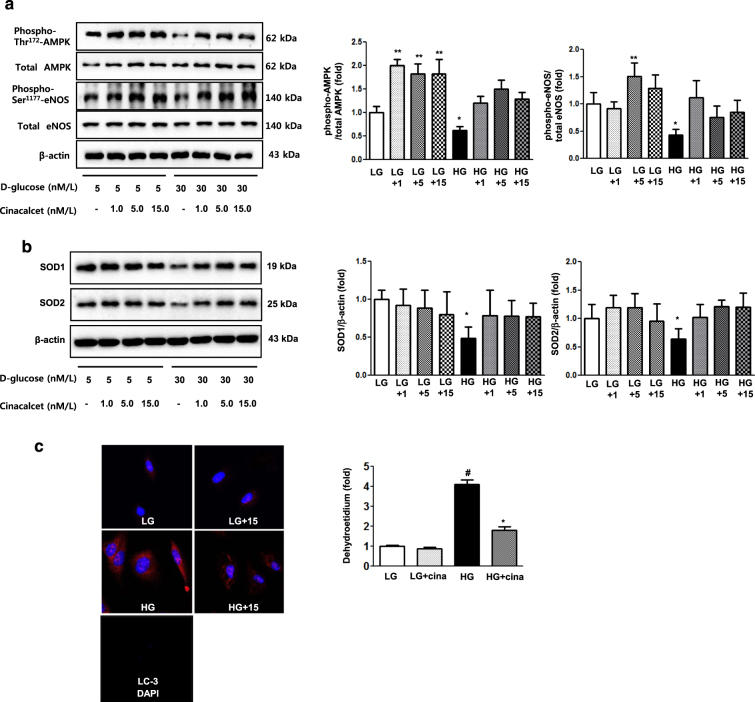

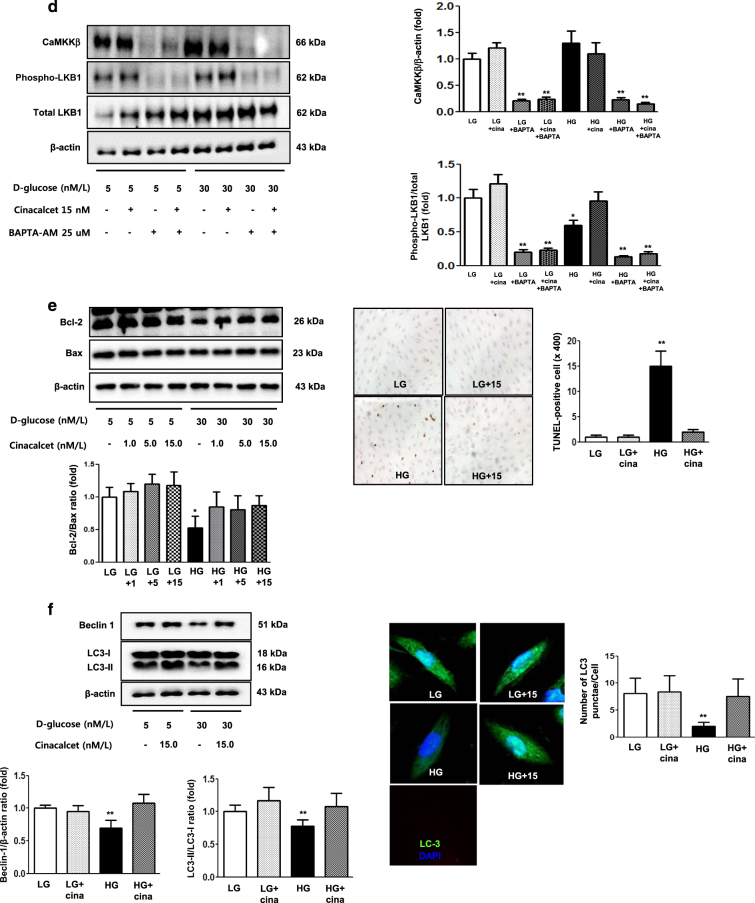
The effect of cinacalcet on intracellular signaling for AMPK-eNOS oxidative stress and apoptosis in the HGECs cultured in low-glucose (LG; 5 mmol/l D-glucose) or high-glucose (HG; 30 mmol/l D-glucose) conditions with or without cinacalcet treatment (1, 5, 15 nM) (**a–d**). Representative Western blot analyses and quantitative analyses of total AMPK, phosphor-Thr^172^ AMPK, total eNOS, phospho-Ser^1177^ eNOS (**a**, *P < 0.05 and **P < 0.01 compared with LG control), SOD1 and SOD2 (**b**, *P < 0.05 compared with other groups), dihydroethidium expression (as an oxidative stress marker; **c**, *P < 0.05 and #*P* < 0.001 compared with other groups), Bcl-2, Bax, and TUNEL-positive HGECs (**e**, *P < 0.05 and **P < 0.01 compared with other groups), and β-actin levels in the cultured HGECs and their quantitative analyses of the results are shown (*n* = 4 independent experiments in each experiments). **d** The effect of BAPTA-AM (25 μM) on cinacalcet-indueced in the HGECs cultured in low-glucose or high-glucose (HG; 30 mmol/l D-glucose) with or without cinacalcet treatment (15 nM). Representative Western blot analyses and quantitative analyses of CaMKKβ, phospho-LKB1, and total LKB1 (*n* = 4 independent experiments in each experiments). *P < 0.05 and **P < 0.01 compared with LG control. **f** The changes of intracellular signaling related to autophagy in HGECs exposed to cinacalcet and high-glucose media. Representative Western blot analyses and quantitative analyses of beclin-1, LC3-II/LC3-I ratio, and β-actin levels in the cultured HGECs and their quantitative analyses of the results are shown (*n* = 4 independent experiments in each experiments). Representative immunofluorescent analyses of LC3 punctae in HGECs and the quantitative analyses of the results are shown (*n* = 6 independent experiments in each experiments). **P < 0.01 compared with other groups

As hyperglycemia-induced oxidative stress, glomerular cell apoptosis, and insufficient autophagy comprise the hallmark of diabetic alterations in the kidney, we evaluated the effects of cinacalcet on high glucose-induced oxidative stress and on apoptosis with relevance to the CaMKKβ-AMPK-eNOS signaling in cultured HGECs. Immunofluorescence and western blot results demonstrated that high glucose (30 mmol/l of d-glucose) decreased the expression of CaSR, CaMKKβ, phospho-Ser^428^ LKB1 and phospho-Thr^172^ AMPK, (but not of CaMKKα), which were ameliorated by cinacalcet treatment (15 nM) (Fig. 1b). We used 15 nM of cinacalcet, because increasing the concentration above 10 nM did not lead to a further activation of ERK1/2 and other intercellular signaling. Furthermore, a single dose of 25 mg cinacalcet reaches serum cinacalcet level of 14–28 nM in end-stage renal disease patients^28^. Western blot analyses showed that high glucose-induced oxidative stress as reflected by decreased levels of phospho-Thr^172^ AMPK, phospho-Ser^1173^eNOS was ameliorated by cinacalcet treatment (1, 5, 15 nM) (Fig. 2a). Cinacalcet treatment in high-glucose media increased BCL-2/BAX expression and decreased apoptotic GECs when compared with those in high-glucose media alone, which were associated with decreased expression of SOD1, SOD2 and DHE (Figs. 2b, c, e). Furthermore, decreased beclin-1 and LC3-II/LC3-I ratio and the number of LC3-II punctae in high-glucose media were increased to the levels present in low-glucose media with cinacalcet treatment (Fig. 2f). To investigate whether cinacalcet-induced [Ca^2+^]i caused increased expression of CaMKKβ and/or LKB1 phosphorylation in GECs, we used [Ca^2+^]i chelator BAPTA-AM. Interestingly, cinacalcet-induced CaMKK expression and LKB1 phosphorylation were inhibited by BAPTA-AM in both low- and high-glucose media (Fig. 2d). These results suggest that cinacalcet-induced [Ca^2+^]i might activate both CaMKKβ and LKB1 independently.

**Fig. 3:**
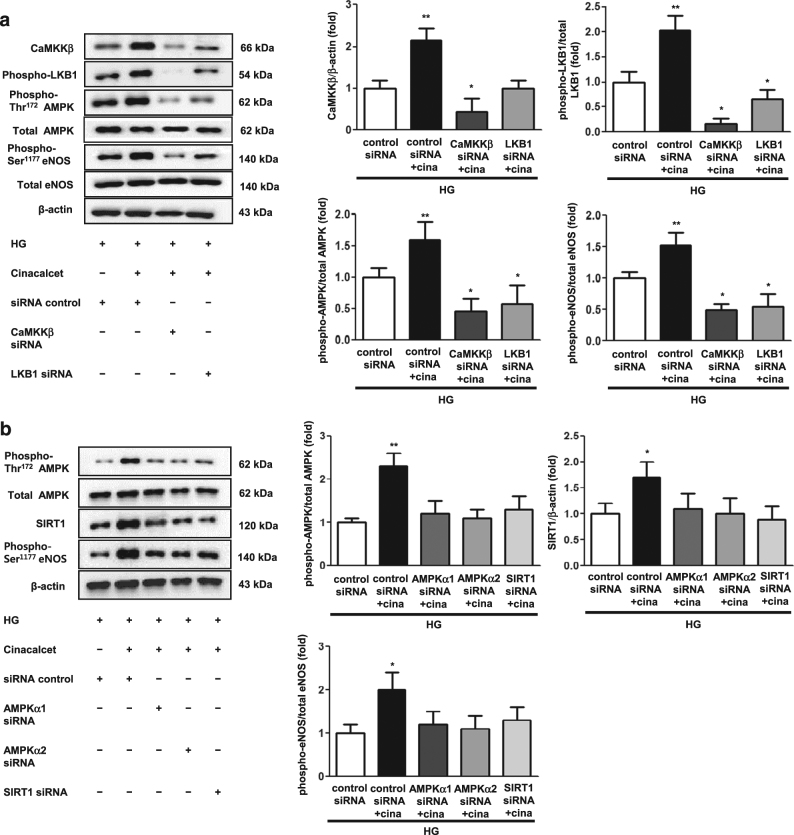
Immunoblot for CaMKKβ, LKB1, phospho-AMPK, SIRT1 and phospho-Ser^1177^ eNOS in AMPKα1 siRNA, AMPKα2 siRNA, or SIRT1 siRNA knock-down HGECs in a high-glucose environment with cinacalcet treatment (**a** and **b**). The cultured HGECs were transfected with a final concentration of 50 nM CaMKKβ and LKB1, α1 and α2-AMPK, SIRT1 siRNAs for 24-h by transfection reagent and treated with cinacalcet (15 nM) in high-glucose media. Representative Western blot analyses of CaMKKβ and phospho-Ser^428^ LKB1 (**a**), as well as phospho-Thr^172^ AMPK, total AMPK, SIRT1, phospho-Ser^1177^ eNOS (**b**) and β-actin levels and the quantitative analyses of the results are also shown (**a** and **b**, respectively) (*n* = 4 independent experiments in each experiments).*P < 0.05, **P < 0.01 compared with control siRNA with HG

To evaluate whether AMPK is involved in the downstream signaling of cinacalcet stimulation, we performed an additional study using siRNAs for *CaMKKβ, LKB1, AMPKα1, AMPKα2*, and *SIRT1* in cultured HGECs. Transfection with *CaMKKβ, LKB1, AMPKα1, AMPKα2*, and *SIRT1* siRNAs suppressed cinacalcet-induced AMPK–SIRT1-eNOS signaling as compared with those of cinacalcet treated siRNA control group in high-glucose media (Figs. 3a, b). These results indicate that cinacalcet effect was mediated predominantly by [Ca^2+^]i-induced CaMKKβ and LKB1 activation and their downstream metabolic regulator, AMPK.

### Increased [Ca^2+^]i by cinacalcet activates CaMKKβ-LKB1-AMPK-eNOS pathway in podocytes

**Fig. 4:**
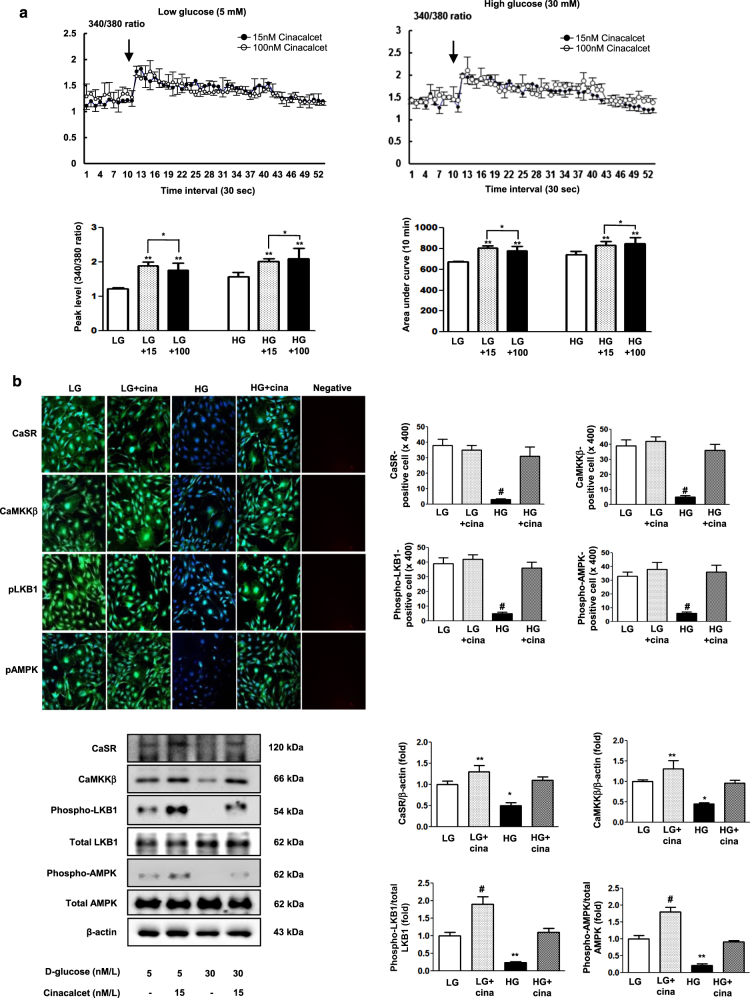
The changes of [Ca^2+^]i and intracellular signaling in podocytes exposed to cinacalcet and high-glucose media. **a** The changes of [Ca^2+^]i in podocytes exposed to cinacalcet and high-glucose media. To determine whether the addition of cinacalcet might modulate [Ca^2+^]i in podocytes, FURA-2AM-loaded podocytes were stimulated using different concentrations (15, 100 nM) of cinacalcet in low-glucose (LG; 5 mmol/l D-glucose) or high-glucose (HG; 30 mmol/l D-glucose) media. The AUC was estimated from the baseline of normalized data (at the point of injection) to a fluorescence level and between time points of injection (0 min) and 10 min. The peak of the curve was measured as highest value of the curve. The peak amplitude and AUC of [Ca^2+^]i were significantly increased by cinacalcet in dose-dependent manners in both LG and HG media. In Fig. 4a, the arrow denotes the administration of cinacalcet (15 and 100 nM, respectively) (*n* = 6 independent experiments in each experiments). **P < 0.01 compared with LG and HG and *P < 0.05 compared with LG + 15 and HG + 15. **b** The changes of intracellular signaling in podocytes exposed to cinacalcet and high-glucose media. Representative immunofluorescent (*n* = 6 independent experiments in each experiments) and western blot analyses (*n* = 4 independent experiments in each experiments) of CaSR, CaMKKβ, phosphor-Ser^428^ LKB1, and phospho-Thr^172^ AMPK in the cultured podocytes in low-glucose (LG; 5 mmol/l D-glucose) or high-glucose (HG; 30 mmol/l D-glucose) conditions with or without cinacalcet treatment (15 nM) and the quantitative analyses of the results are shown (*n* = 6 independent experiments in each experiments). *P < 0.05; **P < 0.01 and #*P* < 0.001 compared with other groups

While endothelial cell injury usually occurs at earlier stages of DN, podocyte injury may be accelerated with or without its influence on GECs. Therefore, we investigated whether the addition of cinacalcet might modulate [Ca^2+^]i in podocytes. FURA-2AM-loaded podocytes were also stimulated using different concentrations (15, 100 nM) of cinacalcet in low and high-glucose media. As shown in Fig. 4a, cinacalcet markedly enhanced [Ca^2+^]i in high-glucose media as compared with low-glucose media in the absence of extracellular calcium. Interestingly, cinacalcet significantly increased the AUC and peak amplitude of [Ca^2+^]i in a dose-independent manner in both low and high-glucose media.

**Fig. 5:**
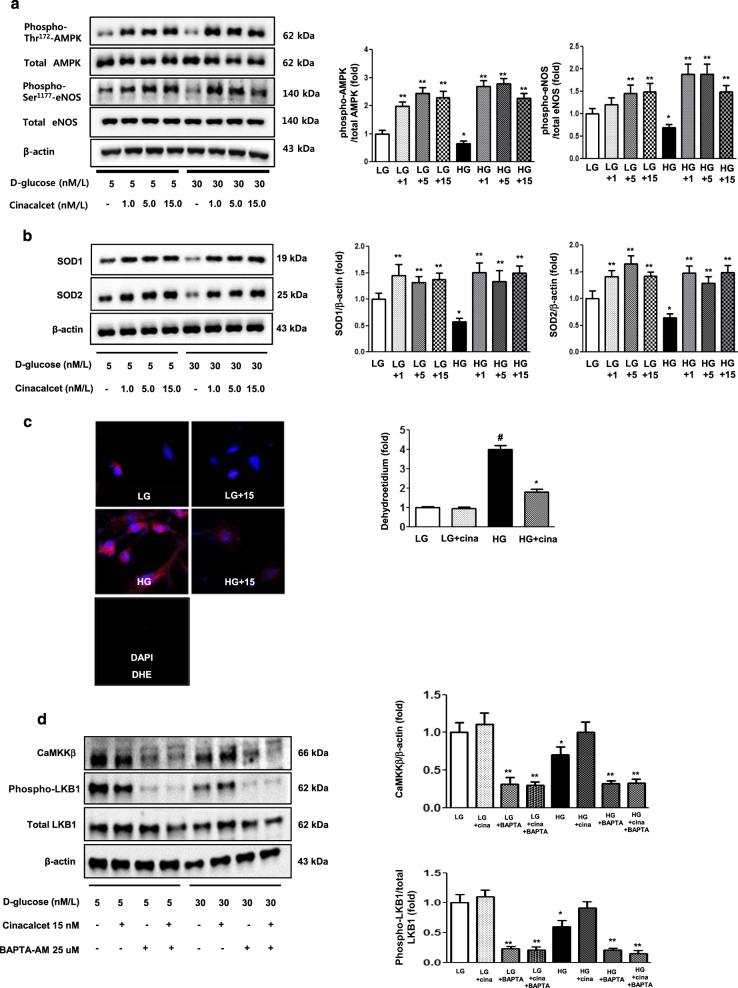

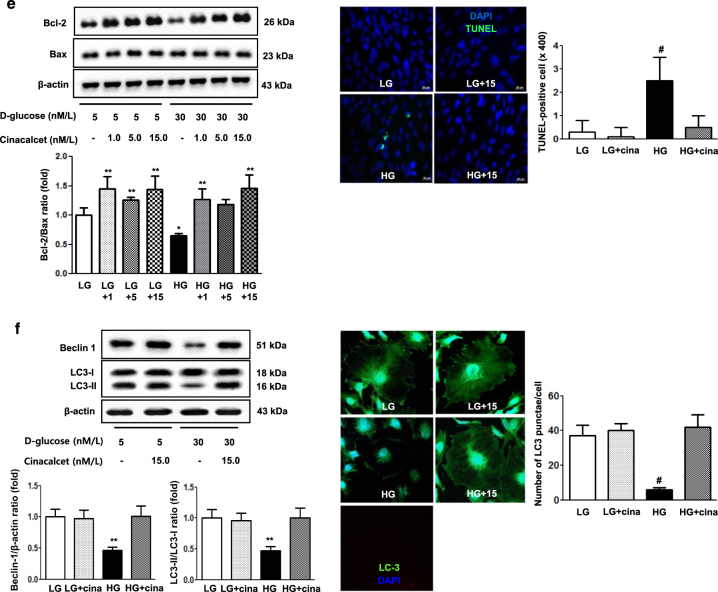
The effect of cinacalcet on intracellular signaling for AMPK-eNOS, apoptosis, and oxidative stress in the podocytes cultured in low-glucose (LG; 5 mmol/l D-glucose) or high-glucose (HG; 30 mmol/l D-glucose) conditions with or without cinacalcet treatment (1 nM, 5 nM, 15 nM) (**a–d**). Representative Western blot analyses and quantitative analyses of total AMPK, phosphor-Thr^172^ AMPK, total eNOS, phospho-Ser^1177^ eNOS (**a**, *P < 0.05 and **P < 0.01 compared with LG control), SOD1 and SOD2 (**b**, *P < 0.05 and **P < 0.01 compared with LG control), dihydroethidium expression (**c**, *P < 0.05 and #*P* < 0.001 compared with other groups), Bcl-2, Bax, and TUNEL-positive podocytes (**e**, *P < 0.05 and **P < 0.01 compared with LG control and #*P* < 0.001 compared with other groups), and β-actin levels in the cultured podocytes and their quantitative analyses of the results are shown (*n* = 4 independent experiments in each experiments). **d** The effect of BAPTA-AM on cinacalcet-indueced in the podocytes cultured in low-glucose or high-glucose (HG; 30 mmol/l D-glucose) with or without cinacalcet treatment (15 nM). Representative Western blot analyses and quantitative analyses of CaMKKβ, phospho-LKB1, and total LKB1 (*n* = 4 independent experiments in each experiments). *P < 0.05 and **P < 0.01 compared with LG control. **f** The changes of intracellular signaling related to autophagy in podocytes exposed to cinacalcet and high-glucose media. Representative western blot analyses and quantitative analyses of beclin-1, LC3-II/LC3-I ratio, and β-actin levels in the cultured podocytes and their quantitative analyses of the results are shown (*n* = 4 independent experiments in each experiments). Representative immunofluorescent analyses of LC3 punctae in podocytes and the quantitative analyses of the results are shown. **P < 0.01 and # *P* < 0.001 compared with other groups (*n* = 6 independent experiments in each experiments)

We also evaluated the effects of cinacalcet on high-glucose-induced oxidative stress, apoptosis and autophagy with relevance to the CaMKKβ-AMPK-eNOS signaling in cultured podocytes. Consistent with the findings in GECs, immunoflorescence and western blot results demonstrated that high-glucose decreased the expression of CaSR, CaMKKβ, phospho-Ser^428 ^LKB1 and phospho-Thr^172^ AMPK (but not CaMKKα), which were ameliorated by cinacalcet treatment (15 nM) (Fig. 4b). Western blot and DHE immunofluorescence analyses showed that high-glucose-induced oxidative stress as reflected by decreased levels of SOD1 and SOD2 and increased expression of DHE were ameliorated by cinacalcet treatment (1, 5, 15 nM) (Figs. 5b, c), which were associated with recovery of phospho-Thr^172^ AMPK and phospho-Ser^1177^ eNOS expression (Fig. 5a). Cinacalcet treatment in high-glucose media increased the expression of BCL-2/BAX and decreased the number of apoptotic podocytes when compared with those in high-glucose media alone (Fig. 5e). Furthermore, decreased beclin-1 and LC3-II/LC3-I ratio and the number of LC3-II punctae in a podocyte in high-glucose media were increased to the levels present in low-glucose media with cinacalcet treatment (Fig. 5f). We then investigated whether cinacalcet-induced increase in [Ca^2+^]i enhanced the expression of CaMKKβ and/or LKB1 phosphorylation in podocytes as well. Cinacalcet-induced CaMKK expression and LKB1 phosphorylation were inhibited by BAPTA-AM in both low and high-glucose media (Fig. 5d). These results suggest that cinacalcet-induced [Ca^2+^]i might activate both CaMKKβ and LKB1 independently in podocytes as well.

### Cinacalcet ameliorates diabetes-induced albuminuria and hypercalciuria

Body weight, kidney weight, blood glucose concentration, and HbA1c level were significantly higher in *db/db* mice than in that of *db/m* mice, regardless of cinacalcet treatment (Table 1). There were no differences in the serum creatinine and Ca^2+^ levels among all study groups. Albuminuria, creatinine clearance (Ccr) and urinary calcium/creatinine ratio increased significantly in *db/db* mice compared with those in *db/m* mice treated with or without cinacalcet. However, cinacalcet treatment ameliorated albuminuria, Ccr and urinary calcium/creatinine ratio to the levels present in *db/m* mice (Table 1).

**Table 1:** Biochemical and physical characteristics of all study groups

	*dm* cont	*dm* + cina	*db* cont	*db* + cina
Body wt (g)	30.9 ± 1.9	32.3 ± 2.2	59,8 ± 5.7**	55.9 ± 9.4**
Kidney wt/Body wt (g/g)	0.005 ± 0.015	0.005 ± 0.013	0.004 ± 0.003	0.003 ± 0.003
BUN (mg/dL)	16.2 ± 2.9	18.5 ± 2.9	18.2 ± 1.2	20.8 ± 5.0
Cr (mg/dL)	0.076 ± 0.010	0.077 ± 0.008	0.078 ± 0.011	0.076 ± 0.009
HbA1c (%)	4.6 ± 0.2	4.4 ± 0.4	13.0 ± 1.2**	12.2 ± 1.3**
HbA1c (mmol/mol)	27 ± 0.91	25 ± 1.82	119 ± 5.46**	110 ± 5.91**
Glucose (mg/dL)	230 ± 28	187 ± 16	577 ± 49**	534 ± 84**
iCa^++^ (mmol/L)	1.28 ± 0.02	1.20 ± 0.07	1.32 ± 0.05	1.27 ± 0.06
PO4^−^ (mmol/L)	3.9 ± 0.7	4.0 ± 0.8	4.1 ± 1.0	4.0 ± 0.9
24 h albuminuria (ug)	12.7 ± 7.9	14.1 ± 6.0	289.8 ± 104**	121.0 ± 45.9*
Ccr (ml/min)	0.33 ± 0.19	0.31 ± 0.27	0.58 ± 0.22**	0.39 ± 0.23
Urine Ca/Cr ratio	0.04 ± 0.02	0.03 ± 0.01	0.14 ± 0.06^#^	0.06 ± 0.03

*Ca* total calcium, *Cr* creatinine, *iCa*^*++*^ ionized Ca^++^

*P < 0.05 ; **P < 0.01; ^#^*P < *0.001 compared to other groups (*n* = 8 in each experiment)

### Cinacalcet ameliorates diabetes-induced renal damage by reducing intrarenal inflammation

**Fig. 6:**
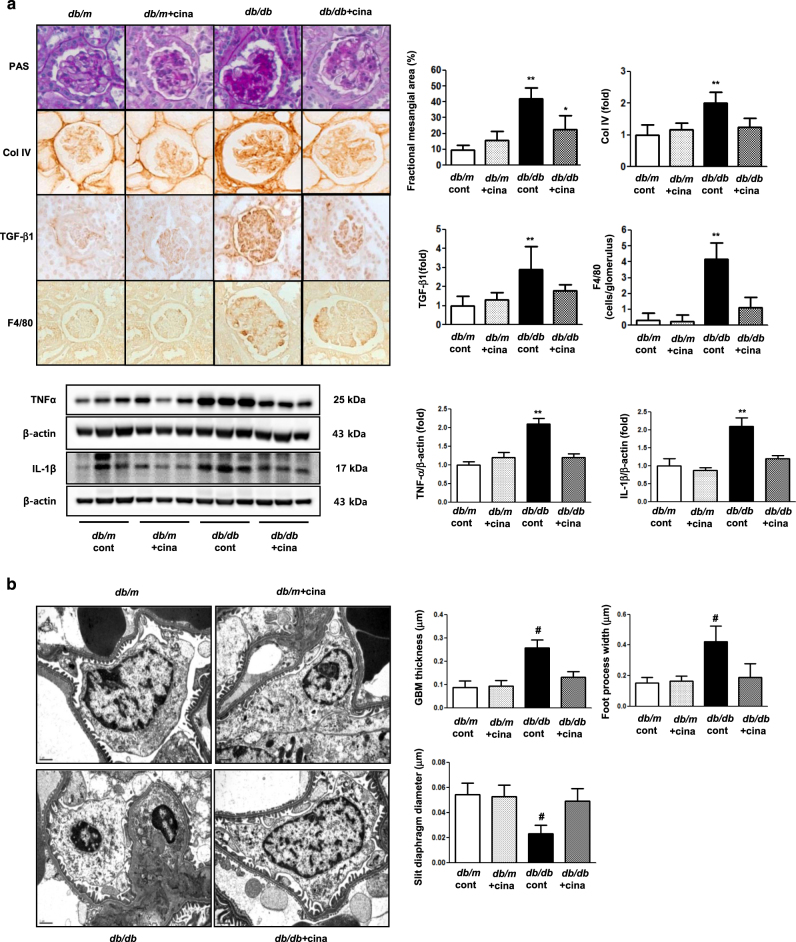
Effects of cinacalcet on renal phenotypes in *db/m* and *db/db* mice at 20 weeks. **a** Glomerular mesangial fractional area, TGF-β1 and type IV collagen (Col IV) expression, and F4/80-positive cell infiltration in the glomerulus in the cortical area of the *db/m* and *db/db* mice, with or without cinacalcet treatment. Representative sections stained with periodic acid-Schiff reagent and representative immunohistochemical staining for TGF-β1, Col IV, and F4/80-positive cells (dark brown) are shown (original magnification, x400). Quantitative analyses of the results for the mesangial fractional area (%), TGF-β1, COL IV, and F4/80-positive cells (fold) are shown (*n* = 8 in each groups). Representative Western blot analyses of the TNF-α, IL-1β, and β-actin expressions and their quantitative analyses of the results are shown (*n* = 4 independent experiments in each experiments). *P < 0.05 and **P < 0.01 compared with *db/m* cont and *db/m* + cina groups. **b** Effects of cinacalcet on podocyte phenotypes in *db/m* and *db/db* mice at 20 weeks. The thickened glomerular basement membrane and widened foot process width and narrowed slit diaphragm diameter in *db/db* mice compared with *db/m* mice were normalized after cinacalcet treatment (*n* = 8 in each groups). # *P* < 0.001 compared with *db/m* cont and *db/m* + cina groups

We estimated the extent of mesangial expansion (by measuring fractional mesangial area) to determine the degree of glomerular functional deterioration and to predict the severity of glomerular lesions in diabetic nephropathy. There were no significant differences in fractional mesangial area between *db/m* mice treated with or without cinacalcet (Fig. 6a). In contrast, there was a significant increase in the mesangial areas of *db/db* mice which was ameliorated by cinacalcet administration (*P < *0.01). Consistent with the changes in the mesangial fractional area, increased expression of the pro-fibrotic growth factor TGF-β1, extracellular matrix Col IV, and increased glomerular inflammatory cell infiltration in *db/db* mice were decreased by cinacalcet treatment (Fig. 6a). Cinacalcet treatment also decreased such proinflammatory cytokines as TNFα and IL-1β in the kidneys (Fig. 6a). The thickened glomerular basement membrane, widened foot process width, and narrowed slit diaphragm diameter in *db/db* mice were ameliorated by cinacalcet treatment (Fig. 6b).

### **Cinacalcet ameliorates oxidative stress through the activation of the CaSR-CaMKKβ-LKB1-AMPK-eNOS pathway in the kidney**

**Fig. 7:**
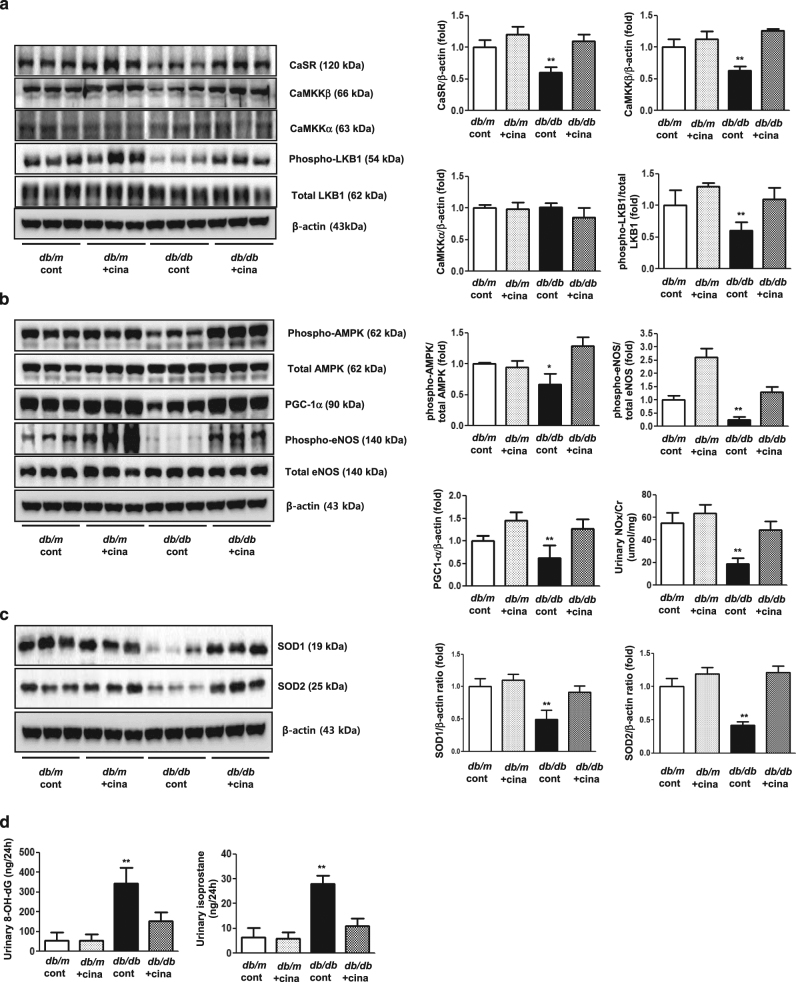

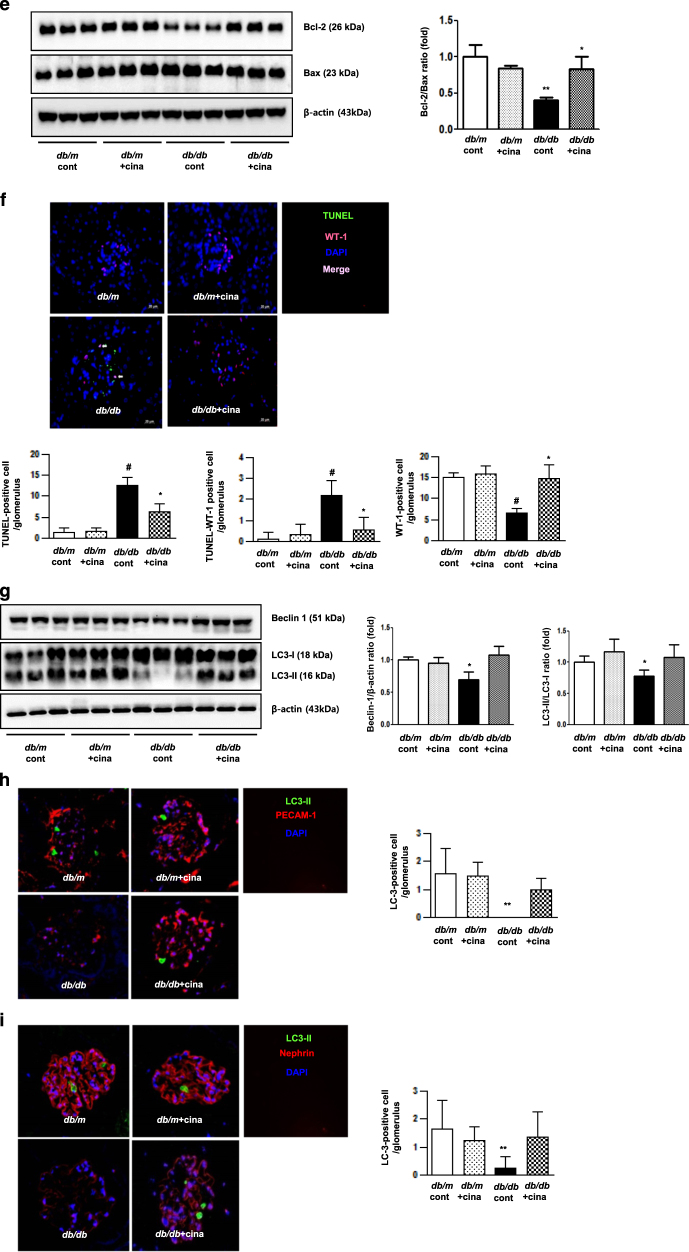
Intra-renal expressions of the CaSR and CaMKKβ-LKB1-AMPK-eNOS signaling pathways in *db/m* and *db/db* mice without or with cinacalcet treatment. **a** and **b** Representative Western blot analyses of the CaSR, CaMMKα/β, total LKB1, phospho-Ser^428^ LKB1, total AMPK, phospho-Thr^172^ AMPK, PGC-1α, total eNOS, phospho-Ser^1177^ eNOS and β-actin expressions. Quantitative analyses of the results are shown (*n* = 3 in each experiments). *P < 0.05 and **P < 0.01 compared with other groups. **c** Intra-renal expressions of the SOD1 and SOD2 and **d** 24-h urinary 8-hydroxy-deoxyguanosine (8-OH-dG) and isoprostane concentrations in *db/m* and *db/db* mice without or with cinacalcet treatment. Representative Western blot analyses of the SOD1 and SOD2 (*n* = 3 in each groups) and 24-h urinary 8-OH-dG and isoprostane concentrations of the results are shown (*n* = 8 in each experiments). *P < 0.05 and **P < 0.01 compared with other groups. **e** Intra-renal Bcl-2/Bax ratio and **f** TUNEL-and WT-1-positive cells in the glomerulus in *db/m* and *db/db* mice without or with cinacalcet treatment. Representative Western blot analysis of the proapoptotic Bax, antiapoptotic Bcl-2 and β-actin expressions (*n* = 3 in each experiments) and immunohistochemical staining for TUNEL-positive cells (dark brown) are shown (original magnification, x400) (*n* = 8 in each groups). The quantitative analyses of the results are shown. *P < 0.05; **P < 0.01, and #*P* < 0.001 compared with other groups. **g** Intra-renal beclin 1 and LC3-II/LC3-I ratio and **h** LC3-II-PECAM-1, and **i** LC3-II-nephrin-positive cell in the glomerulus of *db/m* and *db/db* mice without or with cinacalcet treatment. Representative Western blot analysis of beclin-1, LC-3-I, LC3-II, and β-actin expressions are shown. The quantitative analyses of the results are shown (*n* = 3 in each groups). *P < 0.05 and **P < 0.01 compared with other groups

Cinacalcet treatment markedly increased the expression of intrarenal CaSR, CaMKKβ, phospho-Ser^428^ LKB1, phospho-Thr^172^ AMPK, PGC-1α, and phospho-Ser^1177^eNOS in *db/db* mice. There were no differences in the levels of CaMKKα, phospho-Thr^172^ AMPK among *db/m* mice treated with or without cinacalcet (Fig. 7a,b).

### Cinacalcet exerts anti-oxidative, anti-apoptotic, and pro-autophagic effects

It is well known that eNOS phosphorylation leads to anti-oxidative, anti-apoptotic and pro-autophagic activities by enhancing NOx and the BCL-2 activity and down-regulating the pro-apoptotic BAX activity. Cinacalcet-induced phosphorylation of eNOS increased urinary NOx (Fig. 7b), renal SOD1, and SOD2 levels (Fig. 7c) and decreased urinary 8-hydroxy-deoxyguanosin and isoprostane concentrations, reflecting a net decrease in oxidative stress in the kidney (Fig. 7d). Moreover, cinacalcet decreased the expression of BAX protein while increasing that of BCL-2 protein in *db/db* mice (Fig. 7e, *P < *0.01). Consistently, increased expression of BCL-2/BAX ratio, reduced number of TUNEL-positive cells and TUNEL-positive WT-1 cells in the glomerulus with cinacalcet treatment collectively increased the number of functioning podocytes (Fig. 7f, *P < *0.01). Decreased expression of beclin-1 and LC3-II/LC3-1 ratio in *db/db* mice, indicating a reduced autophagy initiation, was also recovered to the levels present in *db/m* with cincalcet treatment (Fig. 7g), especially in HGECs (Fig. 7h) and murine podocytes (Fig. 7i).

## Discussion

This study demonstrated that, (in cultured HGECs and podocytes in high-glucose media), cinacalcet-induced increase in [Ca^2+^]i phosphorylates CaMKKβ/LKB1, and subsequently activates AMPK and their downstream targets including eNOS, which overall ameliorates oxidative and apoptotic stress and enhances autophagy process. Furthermore, the study of HGECs transfected with *Ampkα1*, *Ampkα2*, *Sirt1* and *CaMKKβ* siRNAs in high-glucose media confirmed that cinacalcet-induced favorable renal effects were rendered through the activation of AMPK-eNOS signaling. It is well known that eNOS is one of the direct targets of AMPK activation, which exerts renoprotective effect^26,29^, especially in DN. Cinacalcet ameliorated albuminuria in *db/db* mice without influencing blood glucose and Ca^2+^ concentrations. Diabetes-induced renal phenotypic alterations and the extent of inflammatory cytokines and inflammatory cell infiltration in the glomerulus were also restored by cinacalcet treatment. At the molecular level, cinacalcet increased the expression of CaSR and phosphorylation of AMPK by activating its upstream kinases CaMKKβ-LKB1, which subsequently activated PGC-1α, phospho-Ser^1177^ eNOS-NO signaling and increased BCL-2/BAX ratio in renal cortex. Moreover, cinacalcet decreased the levels of urinary 8-hydroxy-deoxyguanosin and isoprostane, while increasing the expression of such antioxidant enzymes as SOD1 and SOD2. Impaired AMPK activity with resultant ROS generation, enhanced initiation of the apoptosis, and of autophagic damage is implicated in DN. Interestingly, the fact that cinacalcet could restore diabetes-induced dysfunctions in an animal model and at a cellular level in HGECs and podocytes through the activation of AMPK suggests its therapeutic potential in the prevention and treatment of DN.

The fundamental backdrop of diabetic kidneys in streptozotocin-induced diabetic rats lies in the reduced expression of CaSR (up to 52% of non-diabetic controls) and ascribes this phenomenon to the altered divalent cation homeostasis which can be presented as hypercalciuria^30^. Our study demonstrated that cinacalcet significantly upregulates CaSR expression in the kidneys of *db/db* mice, and this upregulation has a potential reinforcing effect on [Ca^2+^]i homeostasis and subsequent AMPK activation by modulation of CaMKKβ and LKB1. Pre-exposure of GECs and podocytes to the BAPTA-AM, an [Ca^2+^]i chelator, prevented cinacalcet-induced CaMKKβ and LKB1 activation. These results suggest that cinacalcet-induced CaMKKβ and LKB1 activation might be related to an increase in [Ca^2+^]i. Recent study showed that the LKB1/CaMKK-AMPK axis and [Ca^2+^]i levels play a critical role in anchorage-independent cancer sphere formation. Thus, the Ca^2+^/reactive oxygen species-triggered LKB1/CaMKKβ-AMPK signaling cascade may provide a quick, adaptable switch to promote survival of metastasizing cancer cells^31^. Moreover, stimulation of 3T3L1 adipocytes with [Ca^2+^]i raising agents results in an activation of the AMPK pathway^32^. Ma et al. also showed that treatment of baicalin, a flavone, causes an increase in [Ca^2+^]i concentration at the similar amount to our result, which activated CaMKKβ in LKB1 deficient HeLa and A549 cell line^33^. In contrast, increased [Ca^2+^]i can also activate other protein kinases, which potentially affect apoptosis and autophagy of GECs and podocytes related to endoplasmic reticulum stress and mitochondrial dysfunction, such as calcium/calmodulin dependent protein kinase II^34^. The precise mechanism underlying CaSR upregulation by cinacalcet and subsequent CaMKKβ/LKB1 activation is not completely understood. Therefore, further studies are warranted to elucidate cinacalcet-mediated upregulation of CaSR expression in a diabetic milieu.

It has been well known that a loss of AMPK activity or attenuation of its expression leads to significant metabolic disorders including diabetic complications^29^. Until now, it is believed that most of the existing agents activate AMPK indirectly by inhibiting ATP production, whether by restraining oxidative phosphorylation (metformin, thiazolidinediones, resveratrol, and berberin) or by glycolysis (2-deoxyglucose), thus increasing cellular ADP:ATP and AMP:ATP ratios^21^. According to the present study, cinacalcet at a concentration of 1.0–15 nM/L, that is comparable to the physiologic serum concentrations of uremic hyperparathyroidism patients^28^, significantly increased [Ca^2+^]i levels by mobilizing intracellular Ca^2+^ stores in HGECs cultured in Ca^2+^-free media. This finding has been observed in response to depolarization of neurons^35^ and in T lymphocytes^30^ in the absence of any changes in cellular nucleotides^36^. In vitro studies of HGECs and podocytes with low-glucose or high-glucose media indicated that cinacalcet markedly enhanced [Ca^2+^]i in the absence of extracellular calcium in a dose-dependent manner. Independent of adenylated energy balance, such as the ratios of AMP:ATP and ADP:ATP, either of the two upstream kinases, CaMKKβ or LKB1, has been shown to phosphorylate and activate AMPK in response to increased [Ca^2+^]i^37,38^. Our study is the first to present cinacalcet-induced increase in [Ca^2+^]i with subsequent increase in the expression of CaMKKβ and LKB1 phosphorylation and activation of downstream signaling including AMPK-PGC-1α-eNOS in DN, GECs, and podocytes in particular. These results suggest that cinacalcet directly activates AMPK without affecting cellular nucleotide levels in the GECs and podocytes. Thus, it is serum [Ca^2+^]i that acts upon upstream target molecules, CaMKKβ and LKB1, to activate AMPK.

Along with our previous reports^24,25^, other studies^39–41^ have also demonstrated decreased AMPK activity in diabetic conditions. Reduced AMPK activity is associated with increased mTOR activity and resultant renal hypertrophy in DN^41^. One recent study revealed that hyperglycemia contributes to the reduction of LKB1 activity as well. Interestingly, resveratrol treatment reversed the adverse effects of hyperglycemia through the activation of AMPK and LKB1^42^. AMPK activation in endothelium prevents inflammation^43,44^ and promotes fatty acid oxidation^45^, angiogenesis^46^, and NO production^47^. Overall, the effect of AMPK activation in endothelium appears to be antiatherogenic, resulting in the improvement of the endothelial dysfunction. In addition to its renoprotective role in diabetic kidney, the current study emphasizes the significant effect of cinacalcet in terms of AMPK activation in improving podocyte dysfunction. Moreover, a renoprotective action of cinacalcet has been recently reported that it exerts pro-survival signaling and genomic actions and stabilizes the actin cytoskeleton in podocytes^48^ and also prevents podocyte loss in uninephrectomized ApoE-null mice^49^. Another study showed that cinacalcet attenuates the renal endothelial-to-mesenchymal transition in rats with adenosine-induced renal failure by reducing serum PTH level^50^. It suggests that cinacalcet could attenuate renal damage through both direct and PTH-dependent pathways. Of interest, there was a discrepancy in cellular apoptotic response between HGECs and podocytes. Previous studies showed that there are big differences in cell susceptibility and in the time course of the cellular damage reaction between these cell types. Regardless of species, GEC dysfunction and damage precede podocyte injury in adriamycin-susceptible mouse strain^51^. Evidence also shows that podocytes have a high basal level autophagic activity which may contribute to enhanced cellular homeostasis^52,53^. We assume that GECs’ innate vulnerability to external stimuli and podocytes’ high basal autophagic activity might explain different apoptotic cell responses being exerted between these two cell types.

Recruitment of inflammatory cells to the glomerulus is a common yet serious feature of DN. Accumulating results support that the reduced activities of AMPK and PGC-1α appear to be linked to the early manifestations of renal inflammation and profibrotic pathways^54^. Characteristically, PGC-1α level is decreased in diabetic kidneys in association with reduced AMPK activity, reduced mitochondrial complex activity, and dysregulated oxidative stress^55^. Therefore, reversal of these dysfunctions might be helpful in the resolution of inflammation in DN. Collectively, the current study showed that cinacalcet treatment can protect endothelial and podocyte inflammation and oxidative stress through enhancement of AMPK-PGC-1α pathway with subsequent potentiation of eNOS signaling^24,56^.

Hypercalciuria is one of the early findings of uncontrolled diabetes in both humans and experimental animals^29,56,57^. It is associated with high glomerular filtration rate with polyuria, increased dietary calcium intake due to hyperphagia and decreased expression of CaSR in distal tubules. Furthermore, it is reported recently that renal loss of α-klotho in the distal convoluted tubules causes urinary calcium excretion in diabetic animal models^58^. The effect of cinacalcet on urinary calcium is not known and difficult to predict, especially in DN. In the current study, we demonstrated that increase in urinary calcium excretion in *db/db* mice was significantly lowered with cinacalcet treatment. In this study, cinacalcet treatment significantly ameliorated glomerular hyperfiltration as reflected by decreased Ccr. It is plausible that urinary calcium excretion may be lowered through the improvement of hyperfiltration and increased expression of CaSR. However, further studies are required to understand how cinacalcet deals with renal tubular calcium handling in diabetic condition.

**Fig. 8:**
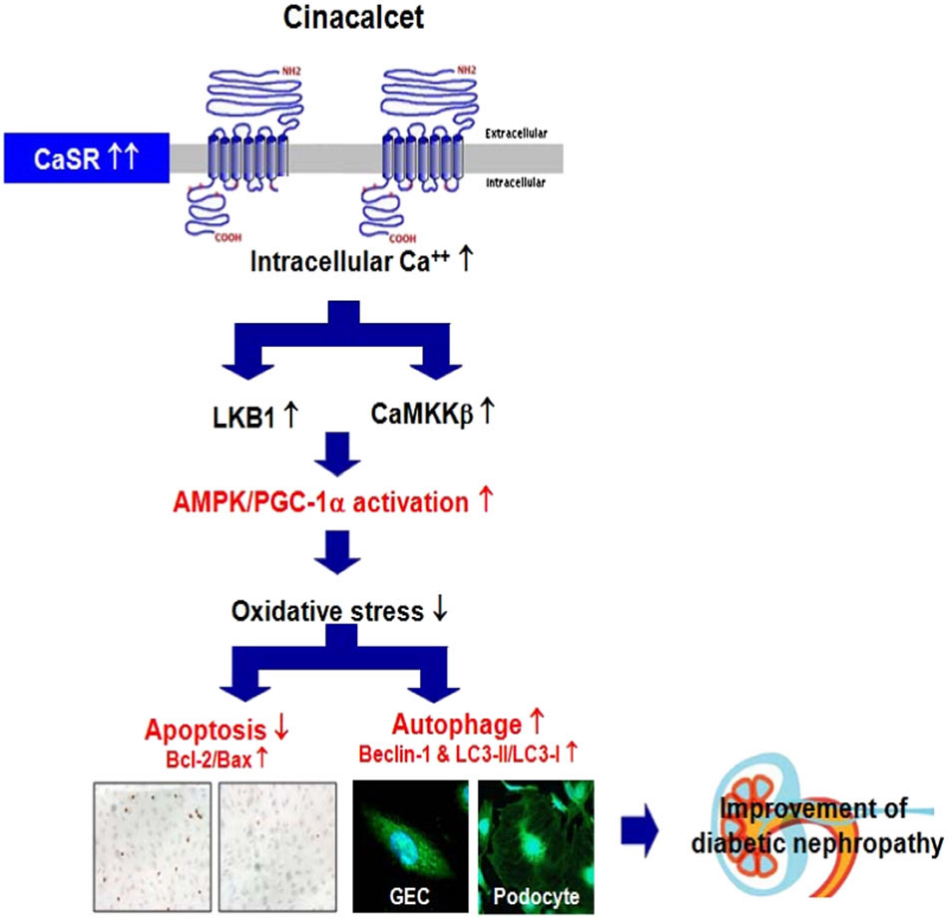
The proposed role of cinacalcet in diabetic nephropathy and the interplay between cinacalcet and kidney, especially glomerular endothelial cells (GECs) and podocytes, in type 2 diabetes. *AMPK* AMP-activated protein kinase, *CaMKKβ* Ca^2+^/calmodulin-dependent protein kinase kinaseβ, *CaSR* calcium-sensing receptor, *LKB1* liver kinase B1, *[Ca*^*2+*^*]i* intracellular calcium, *PGC-1α* peroxisome proliferator-activated receptor γ coactivator 1-α

In conclusion, we would like to highlight that cinacalcet potentially upregulates CaSR expression and activates AMPK through the [Ca^2+^]i-CaMKKβ-LKB1 pathway and subsequent eNOS activation, which ameliorates apoptosis and oxidative stress in GECs and podocytes of diabetic kidney (Fig. 8). Overall, our findings delineate an important physiologic role of [Ca^2+^]i-dependent AMPK activation in DN, indicating that restoring of AMPK activity by cinacalcet holds therapeutic potential for DN.
